# Amiloride resolves resistant edema and hypertension in a patient with nephrotic syndrome; a case report

**DOI:** 10.14814/phy2.13743

**Published:** 2018-06-25

**Authors:** Gitte R. Hinrichs, Line A. Mortensen, Boye L. Jensen, Claus Bistrup

**Affiliations:** ^1^ Department of Cardiovascular and Renal Research University of Southern Denmark Odense Denmark; ^2^ Department of Nephrology Odense University Hospital Odense Denmark; ^3^ Department of Clinical Research University of Southern Denmark Odense Denmark

**Keywords:** ENaC, plasmin, protease, proteinuria

## Abstract

Sodium and fluid retention is a hallmark and a therapeutic challenge of the nephrotic syndrome (NS). Studies support the “overfill” theory of NS with pathophysiological proteolytic activation of the epithelial sodium channel (ENaC) which explains the common observation of suppressed renin –angiotensin system and poor therapeutic response to ACE inhibitors. Blockade of ENaC by the diuretic amiloride would be a rational intervention compared to the traditionally used loop diuretics. We describe a 38‐year‐old male patient with type1 diabetes who developed severe hypertension (200/140 mmHg), progressive edema (of at least 10 L), and overt proteinuria (18.5 g/24 h), despite combined administration of five antihypertensive drugs. Addition of amiloride (5 mg/day) to treatment resulted in resolution of edema, weight loss of 7 kg, reduction in blood pressure (150/100–125/81 mmHg), increased 24 h urinary sodium excretion (127–165 mmol/day), decreased eGFR (41–29 mL/min), and increased plasma potassium concentration (4.6–7.8 mmol/L). Blocking of ENaC mobilizes nephrotic edema and lowers blood pressure in NS. However, acute kidney injury and dangerous hyperkalemia is a potential risk if amiloride is added to multiple other antihypertensive medications as ACEi and spironolactone. The findings support that ENaC is active in NS and is a relevant target in adult NS patients.

## Introduction

Generalized edema is an essential clinical feature of NS independent of various underlying etiologies. Two major hypotheses have been proposed to explain edema formation, the *underfill* and *overfill* theory, respectively, which have been recently reviewed (Ellis 2015; Hoorn and Ellison 2017; Ray et al. 2015; Teoh et al. 2015). In brief, according to the *underfill* theory, the loss of albumin from plasma to the urine leads to reduced plasma oncotic pressure and escape of fluid to the interstitial compartment. The secondary activation of the renin–angiotensin–aldosterone system (RAAS) triggers renal sodium and fluid retention to preserve blood pressure and intravascular volume (Brown et al. 1982b; Oliver and Owings 1960; Usberti et al. 1995; Van de Walle et al. 1996). Data showed that RAAS is not activated in most patients with NS (Brown et al. 1982a; Meltzer et al. 1979); that volume correction with albumin substitution does not resolve edema nor enhances natriuresis (Geers et al. 1984; Koomans et al. 1984) and that RAAS blockade and adrenalectomy (in animals) does not resolve edema (Brown et al. 1984; de Seigneux et al. 2006). The *overfill* theory is based on a primarily impaired Na^+^ excretion in NS kidneys (Ichikawa et al. 1983), recapitulated in isolated *ex vivo* perfused nephrotic kidney. Impaired sodium excretion explains the common coincident suppression of renin and aldosterone in NS and is associated with attenuated effectiveness of, for example, loop diuretics. In agreement, the site of sodium retention along the nephron in NS is after the distal convoluted tubule (Ichikawa et al. 1983). In rat, nephrotic syndrome‐mediated Na^+^ retention was ameliorated by amiloride (Deschenes et al. 2001; Feraille et al. 1993; Lourdel et al. 2005; Svenningsen et al. 2009). Thus, ENaC has been proposed as the culprit in NS although at tissue level ENaC protein abundance and membrane association is not changed dramatically. ENaC has a key role in regulation of the extracellular fluid volume and blood pressure, but besides activation by regulatory hormones, specific proteases can activate the channel (Kleyman et al. 2009; Orce et al. 1980; Passero et al. 2008; Vallet et al. 1997). Proteinuria is associated with urinary serine protease activity primarily due to aberrant filtration of these proteases across a damaged glomerular filtration barrier (Schork et al. 2016; Svenningsen et al. 2009, 2012). Volume retention in NS was prevented by a protease inhibitor (Bohnert et al. 2017). Nephrotic urine activates amiloride‐sensitive current in collecting duct cells. Thus, the *overfill* mechanism is based on proteolytically activated hyperactive ENaC channels as reviewed by Ray et al. (2015).

The diuretic strategy to manage fluid retention in NS is a therapeutic challenge. Loop diuretics are traditionally the drug of choice, with incremental dosing, guided by body weight, GFR, and degree of edema. If this treatment fails to reduce edema, combination with a thiazide diuretic is usually attempted. If insufficient, other strategies have been proposed, for example, intravenous administration of loop diuretics with continuous infusion or in cases of extreme generalized edema or compromised cardiorespiratory function, addition of intravenous human‐albumin or dialysis (Davison et al. 1974; Fliser et al. 1999; Haws and Baum 1993; Weiss et al. 1984). On the other hand, data have clearly shown that loop diuretics are less potent in NS compared to healthy controls (Danielsen et al. 1985; Jespersen et al. 1991) however, active ENaC, which is localized downstream loop diuretic‐sensitive transporters would counteract the effect. At current state of knowledge, data are accumulating to suggest that ENaC blockade with, for example, amiloride is a rational approach to reduce edema and weight in NS based on mechanistic insight (Deschenes et al. 2004; Doucet et al. 2007; Hoorn and Ellison 2017). Two early and empirical attempts to treat nephrotic syndrome with triamterene indicated a beneficial effect on edema (Campanacci et al. 1963; Cavazzuti 1965). A small intervention study showed that amiloride significantly increased natriuresis and reduced weight in nephrotic pediatric patients (Deschenes et al. 2004). No controlled clinical trials in nephrotic patients have been performed, although treatment with amiloride in other conditions with proteinuria have been tested (Andersen et al. 2016; Oxlund et al. 2014; Unruh et al. 2017).

## Case Presentation

**Figure 1:**
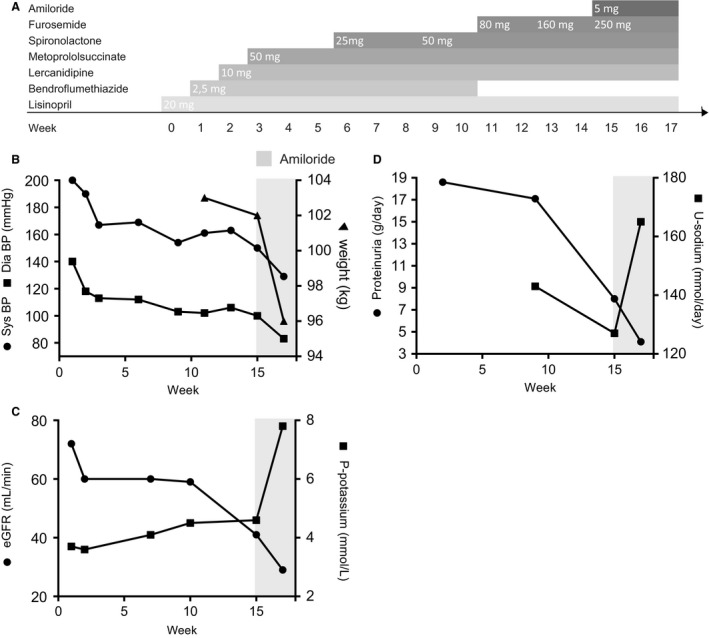
(A): Overview of the increasing doses and numbers of the antihypertensive and diuretic drugs in a timeline. (B) Blood pressure decreased slowly and insufficiently despite several antihypertensive drugs, but at administration of amiloride (week 15), blood pressure, and weight declined promptly and effectively. (C) Combined treatment directed at aldosterone/ENaC resulted in severe hyperkalemia and a decline in kidney function. (D) Addition of amiloride increased urinary sodium excretion. Furthermore, proteinuria decreased.

A 38‐year‐old male was referred to the outpatient clinic at the Department of Nephrology with treatment‐resistant hypertension, rapidly developing edema and overt proteinuria (week 11, Fig. 1A). The patient was initially followed at the outpatient clinic at the Department of Endocrinology with poorly controlled type 1 diabetes for 15 years with microvascular complications including retinopathy and albuminuria, thus presenting with urinary albumin/creatinine ratios over 1000 mg/g for at least 3 years. There were no clinical signs of neuropathy. Plasma creatinine had previously been normal, in the range 60–90 *μ*mol/L. Through several years, the patient had hypertension that was well‐controlled with ACE inhibitors. One year prior to the presentation, blood pressure increased progressively concomitant with development of edema. The patient presented with severe hypertension (200/140 mmHg, week 0, Fig. 1B), edema and urinary protein excretion at 18.5 g/24 h (week 1, Fig. 1D). Despite increasing doses and numbers of antihypertensive agents and diuretics (Fig. 1A), blood pressure continued to be severely elevated combined with progressive fluid overload and proteinuria (Fig. 1B and D). The patient was referred to the Department of Nephrology (week 11, Fig. 1A–D) with NS. At this time, a renography performed on treatment with an ARB revealed no perfusion of the right kidney, and ultrasound confirmed the presence of a 4 cm long, hypoechoic structure in the right retroperitoneal space believed to be a rudimentary right kidney. The left kidney was morphologically and scintigraphically normal. The antihypertensive medication at referral was thiazide, beta‐blocker, calcium channel antagonist, ACE‐inhibitor and mineralocorticoid receptor antagonist spironolactone with no suspicion of noncompliance (Fig. 1A). At presentation, the patient was alert but complained of headache, fatigue, and recent weight gain of 10 kg. On physical examination, blood pressure was 161/102 mmHg, and the patient revealed periorbital and universal pitting edema, no signs of ascites and otherwise normal examination. Based on edema, proteinuria, and hypoalbuminemia (25 g/L), he was diagnosed with NS assumed to be related to diabetes. There was negative test for M‐component and no detectable autoantibodies (ANA, ANCA, anti‐GBA) nor phospholipase A2 antibody. Hepatitis B and C as well as HIV serology were negative. To reduce blood pressure, treatment with a loop diuretic was initiated (furosemide 80 mg/day, Fig. 1A). After 2 weeks without effect on edema and blood pressure (Fig. 1B and D), with a decrease in eGFR and no change in plasma potassium (Fig. 1C), furosemide was stepped up to 160 mg/day (Fig. 1A). Despite treatment with five combined antihypertensive/diuretic agents, there was only a minor weight loss (~1 kg) and reduction in blood pressure. Therefore, a low dose of the ENaC blocker amiloride 5 mg/day was initiated (Fig. 1A) and a 7‐day follow‐up was scheduled, which the patient missed. At next contact after 2 weeks, this treatment resulted in effective resolution of edema, concomitant weight loss of 7 kg and reduction in blood pressure from 150/100 mmHg to 125/81 mmHg (Fig. 1B). The patient continued his normal diet and 24 h urinary sodium excretion increased from 127 mmol/day to 165 mmol/day. Proteinuria decreased from 8 g/day to 4.1 g/day (Fig. 1D), eGFR decreased from 41 mL/min to 29 mL/min and plasma potassium concentration increased from 4.6 to 7.8 mmol/L (Fig. 1C). The patient was immediately hospitalized for cardiac monitoring and treatment of hyperkalemia. Amiloride and spironolactone were both discontinued. At follow‐up 5 weeks later, a combination‐drug containing 2.5 mg amiloride and 25 mg hydrochlorthiazide was successfully reinitiated due to increased blood pressure 141/96 and edema. At the last visit to the outpatient clinic, the patient received the following antihypertensive /diuretic drugs; amiloride/hydrochlorthiazide 2.5 + 25 mg/day, metoprolol 50 mg/day, furosemide 125 mg/day, lercanidipine 10 mg/day, and lisinopril 20 mg/day and his blood pressure was 112/80, body weight was stable at 90 kg (15 kg weight loss), plasma potassium was 4.6 mmol/L, and plasma creatinine was 203 *μ*mol/L with eGFR at 35 mL/min.

## Discussion

The present case illustrates two points; (I) hypertension and edema in NS depends critically on ENaC activity, and (II) when treatment directed at inhibiting aldosterone/ENaC is added on top of other antihypertensives, even with intact kidney function, this involves a serious hazard for hyperkalemia and a decline in kidney function likely related to the abrupt decrease in blood pressure.

The strategy to overcome volume expansion and hypertension in this patient was initially to increase the doses of antihypertensive medication including thiazide/loop diuretic combination. Despite titrating antihypertensives to include five different drugs and notably with high doses of loop diuretics, there was only a very modest reduction in blood pressure and no significant effect on edema. Despite ACE‐inhibition and aldosterone blockade, the powerful action of low‐dose amiloride showed that unopposed ENaC‐mediated Na^+^ transport in the distal nephron (connecting tubules and connecting ducts) is sufficient to compensate for the action of “upstream” loop and thiazide diuretics. Thus the “diuretic resistance” associated with NS is not absolute and could be related to active ENaC. This is in agreement with mouse studies with segmental knock out of ENaC (Perrier et al. 2016) and it is also in agreement with the powerful therapeutic action of systematic, segment‐specific combined diuretics to combat resistant hypertension in patients (Bobrie et al. 2012). The number of ENaC channels expressed at the cell surface is regulated by aldosterone (Masilamani et al. 1999) and the open probability is transitioned to a highly active state by extracellular serine proteases (Caldwell et al. 2005, 2004; Vallet et al. 1997) as recently reviewed (Kleyman et al. 2018). Thus, although MR antagonist would suppress the ENaC abundance in the present patient, these constitutively expressed membrane‐associated channels are likely activated abnormally through proteolysis (Caldwell et al. 2005; Passero et al. 2008; Patel et al. 2012; Picard et al. 2008; Svenningsen et al. 2009). This could explain the sensitivity to amiloride despite spironolactone and ACEi. Amiloride is freely filtered and mainly secreted to the tubular fluid where concentrations 10‐20 *μ*mol/L are achieved (Andersen et al. 2016). Besides blocking ENaC, amiloride inhibits luminal urokinase‐type plasminogen activator (uPA) (Oxlund et al. 2014; Staehr et al. 2015; Vassalli and Belin 1987), a zymogen that converts filtered plasminogen to active plasmin. This may have contributed to the potent natriuretic action, although other serine proteases in the urine may still be active (Svenningsen et al. 2015).

Despite initial dual blockade of RAAS with ACE inhibitors and spironolactone, hyperkalemia did not develop until supplementation with amiloride. This indicates that hyperkalemia was not the result of RAAS inhibition, but rather a result of blocking ENaC. In the current case, hyperkalemia was likely aggravated by the decrease in kidney function which also could explain the reduction in proteinuria. The decline in eGFR was reversible. It could be speculated that the prompt drop in blood pressure in a patient with possible impairment of the autoregulation of renal blood flow combined with a rapid loss of estimated 7L of extracellular fluid might have worsened kidney function. Acute kidney injury and hyperkalemia have previously been reported in a controlled clinical trial in proteinuric patients given amiloride, and the trial was stopped early (Unruh et al. 2017). Thus, the present case and previous studies emphasize the risk of serious adverse effects. This underlines the need to consider exchanging antihypertensives; to monitor potassium concentration and follow blood pressure carefully when initiating amiloride treatment in NS patients who appear highly sensitive. Furthermore, patients should be informed to adhere to low potassium diets and potentially it could be necessary to add a potassium‐binding resin to mitigate the risk of hyperkalemia.

In summary, the present case shows that the addition of the ENaC blocker amiloride to multidrug regimen reduces edema, body weight, and blood pressure in a severe NS case. It also illustrates that careful monitoring and stepwise titration is necessary to minimize the risk for acute injury and hyperkalemia.

## Informed Consent

Written informed consent was obtained from the patient for publication.

## Conflict of Interest

The authors declare that they have no competing interests.
